# Early gross motor development: Agreement between the AIMS and the BSID-III

**DOI:** 10.4102/sajp.v81i1.2168

**Published:** 2025-05-31

**Authors:** Marlette Burger, Esme R. Jordaan, Dana Niehaus

**Affiliations:** 1 Division of Physiotherapy, Department of Health and Rehabilitation Sciences, Faculty of Medicine and Health Sciences, Stellenbosch University, Cape Town, South Africa; 2 Biostatistics Unit, South African Medical Research Council, Cape Town, South Africa; 3 Department of Statistics and Population Studies, University of the Western Cape, Cape Town, South Africa; 4 Department of Psychiatry, Faculty of Medicine and Health Sciences, Stellenbosch University, Cape Town, South Africa

**Keywords:** infants, gross motor development, Alberta Infant Motor Scale, Bayley Scales of Infant Development-III, predictive validity

## Abstract

**Background:**

Early gross motor development is a crucial indicator of overall neurodevelopment. In low- and middle-income countries, lack of accessible assessment tools poses challenges for healthcare professionals evaluating infant neurodevelopment.

**Objectives:**

To determine the agreement between the Alberta Infant Motor Scale (AIMS) and Bayley Scales of Infant Development-III (BSID-III) gross motor domain at 6 months and to evaluate the predictive validity of the AIMS at 6 months for identifying severe gross motor delays at 18 months.

**Method:**

This nested subgroup study assessed 112 full-term infants using both AIMS and BSID-III at 6 months and BSID-III at 18 months. Agreement between measures was determined using Bland-Altman plots, while predictive validity was evaluated using receiver operating characteristic (ROC) curves with various cut-off scores.

**Results:**

Bland-Altman analysis showed strong agreement between AIMS and BSID-III in the lower-performance range, with bias only in scores above 33. The traditional 10th percentile AIMS cut-off had low sensitivity (27.3%) but high specificity (98%) for predicting delays at 18 months. A modified 23rd percentile cut-off improved sensitivity to 63.6% while maintaining acceptable specificity (81.6%), with a 95.2% negative predictive value (NPV).

**Conclusion:**

The AIMS demonstrates strong agreement with BSID-III when identifying potential developmental delays. The proposed 23rd percentile cut-off offers a more balanced screening threshold for this population.

**Clinical Implications:**

The AIMS presents a viable alternative to the BSID-III for initial screening in resource-limited settings. The high NPV at the 23rd percentile cut-off makes it useful for ruling out developmental delays.

## Introduction

The initial 1000 days of life, encompassing a full-term pregnancy of 270 days until the toddler’s second birthday (730 days), is considered the most valuable period to establish the basis for optimum health, growth and neurodevelopment throughout the human’s life (Cusick & Georgieff 2017; Schwarzenberg & Georgieff 2018). The first 1000 days of life are highly significant as no other stage of human life experiences such rapid and profound neurodevelopmental transformation, and the neurodevelopmental processes typically scheduled for this time frame are unlikely to take place later in an individual’s lifespan (Schwarzenberg & Georgieff 2018). The assessment of neurodevelopment typically involves examining the following core domains, which include motor, language, cognitive, social-emotional and adaptive behaviour (Bellman, Byrne & Sege 2013; Fernald et al. 2017). The attainment of gross motor skills is positively associated with the development of other domains, such as cognitive (Ghassabian et al. 2016; Pereira, Valentini & Saccani 2016; Veldman et al. 2019), language (Oudgenoeg-Paz, Volman & Leseman 2012; Walle & Campos 2014) and adaptive behaviour (Ghassabian et al. 2016). The most frequent early, observable deficit in infants potentially at risk of developing neurodevelopmental disorders is in the area of gross motor development (Noritz et al. 2013). Therefore, the accurate and timely assessment and early facilitation of gross motor skill development are essential for achieving the best possible developmental outcomes since the young brain can still adapt (Cioni, Inguaggiato & Sgandurra 2016).

Gross motor assessments are a fundamental component of the paediatric physiotherapist’s practice. Thus, there is a demand for valid and reliable standardised assessment tools to evaluate early gross motor development. Various assessment tools are available for the early evaluation of neuromotor development, including screening tools and comprehensive measures. Screening tools are brief assessments used to detect whether a child is at risk of exhibiting delays in one or several developmental domains. However, the usefulness of screening tools in evaluating subtle delays that might have a notable effect on later development is limited (Sabanathan, Wills & Gladstone 2015). On the other hand, comprehensive measures assess a wide repertoire of skills and provide information about the child’s developmental delays or strengths, even for skills below or above the specific age bracket of the child (Sabanathan et al. 2015; Semrud-Clikeman et al. 2017). Neurodevelopmental delays are always determined based on the comparison with large representative samples of typical or normative development within a given paediatric population. Hence, it should be noted that cut-off scores used to define developmental delays in one population cannot be automatically applied to determine delays in a different population (Fernald et al. 2009).

The lack of normative data on the achievement of developmental milestones in healthy infants across socio-economic, cultural and ethnic groups is a significant obstacle in evaluating and promoting early childhood development within healthcare systems in countries classified as low- and middle-income (LMICs) (Semrud-Clikeman et al. 2017; Zoumenou et al. 2023). Currently, no standardised, norm-referenced comprehensive measurement tools are available to evaluate the various neurodevelopmental domains of children in South Africa. The Bayley Scales of Infant and Toddler Development (BSID) (Bayley & Aylward 2019) was recommended by the World Bank as the principal instrument for assessing early childhood developmental progress in LMICs (Fernald et al. 2009). The BSID is considered the benchmark assessment tool, and it is currently one of the most commonly used neurodevelopmental tools in LMICs (Fernald et al. 2017). The BSID (I–IV) was designed and norm-referenced in the USA. Although no normative data are available for the BSID in South Africa, research has shown that the BSID-III can be utilised to evaluate infant and toddler motor, cognitive and language development in the local context (Ballot et al. 2017; Rademeyer & Jacklin 2013). However, the BSID is extremely expensive to purchase, time-consuming to conduct and score, and necessitates intensive training to meet reliability standards. Furthermore, only a licensed psychologist has the authority to buy the test from the publishing company (Fernald et al. 2017). As a result, it may not be feasible for physiotherapists to utilise the BSID in clinical settings in South Africa to assess gross motor development. In comparison, the AIMS is inexpensive, requires less time for training, involves limited interaction with the infant, can be conducted with basic resources in a small venue and is easy to administer and score (Piper & Darrah 2021).

The AIMS is a performance-based, observational measurement tool of qualitative and quantitative gross motor skill acquisition from birth (term age) to 18 months post-term and was norm-referenced in Canada (Piper & Darrah 1994, 2021). The AIMS is a psychometrically sound instrument that can measure small improvements or changes in gross motor development (Piper & Darrah 2021). The AIMS has been validated for full-term infants aged 4, 8, and 12 months in Cape Town, South Africa (Manuel, Burger & Louw 2012). The study found that South African infants performed better than Canadian infants at 4 months corrected age. However, when assessed at 8 and 12 months of corrected age, no significant distinction was observed between the two groups (Manuel et al. 2012). The purpose of the current study was to compare the predictive ability of the AIMS to the gold standard, namely the BSID-III, in a group of low-risk infants. The specific aims of the current study were to: (1) establish the agreement between the AIMS and the BSID-III gross motor domain at 6 months and (2) evaluate the sensitivity and specificity of the AIMS at 6 months in predicting severe developmental delay on the BSID-III gross motor domain at 18 months in full-term infants with a low risk for gross motor delays.

## Research methods and design

### Study design and setting

This current study represents a nested subgroup and forms part of a large, longitudinal cohort study known as the ‘Maternal and Infant Mental Health (MIMH) study’. The MIMH study recruited expectant women during their second or third trimester from the Stikland Maternal Mental Health Outpatient Clinic based at a Stikland Psychiatric Hospital in the northern suburbs of Cape Town. Recruited mothers who gave informed consent were monitored prospectively until their children reached 4 years of age. Participating mothers who gave informed consent were either of mixed ancestry (speaking Afrikaans or English) or black African (speaking isiXhosa or English).

### Participants

Infants born between 01 April 2014 and 30 September 2019, to mothers with a psychiatric diagnosis, as specified by the Diagnostic and Statistical Manual of Mental Disorders, Fifth Edition (DSM-V) (American Psychiatric Association 2013), fulfilled the requirements for inclusion in the current study. Additionally, a comparison group of mother–infant pairs residing in the same low-income urban and peri-urban communities as the primary MIMH study cohort was also included in the study. At the 6-month assessment, the study group consisted of 118 mother–infant pairs and included 35 infants with no exposure to maternal mental health disorders (comparison group). At the 18-month assessment, six toddlers were lost to follow-up because the families relocated to other parts of the country. One of the aims of the MIMH study was to explore the associations between persistent maternal mental health disorders at 3, 6 and 18 months and infant cognitive, motor, language, social-emotional and adaptive behaviour at 6 and 18 months corrected age after adjusting for socio-demographic variables and mother-infant health outcomes (Burger et al. 2022). We found no significant gross motor developmental delays at 6 or 18 months in infants and toddlers exposed to maternal mental health disorders. Since infants and toddlers exposed to maternal mental health disorders did not exhibit significant gross motor delays, we consider them a low-risk group in this domain. These findings were published in Burger et al. (2022, 2023).

### Measures and procedures

Research assistants collected comprehensive maternal and infant peri- and postnatal health information from hospital records and the Children’s Road to Health booklets (Western Cape Government 2019) and maternal interviews. Structured interviewer-administered questionnaires were used to collect socio-demographic variables. Details on eligibility criteria and health and socio-demographic characteristics at 6 and 18 months assessments are available in previous publications (Burger et al. 2022, 2023). The data were collected over 8 years, from 01 April 2014 to 31 May 2022. Gross motor evaluations were performed by a trained paediatric physiotherapist (the first author) blinded to the infants’ and toddlers’ risk factors. At 6 months, the BSID-III (Bayley 2006) and the AIMS (Piper & Darrah 1994) were used. Both assessments were conducted by the same physiotherapist (the first author) on the same day during a single session. Rest breaks were provided if infants showed signs of fatigue or hunger, ensuring optimal conditions for assessing gross motor performance. The AIMS assessment and scoring typically took 10–15 min, while the gross motor subscale of the BSID-III usually required 20–30 min to complete.

At 18 months, only the BSID-III was administered. The assessments were conducted in the child’s language of choice (Afrikaans, English or isiXhosa), following the administration and scoring guidelines outlined in the BSID-III and AIMS manuals. Assessments took place in a controlled environment: a quiet, comfortable venue free from distractions, with sufficient space for toddlers to display various gross motor skills like rolling, crawling, walking, running, climbing stairs and jumping. To minimise distractions, the evaluation was conducted with only three individuals present, such as the assessor (the first author), the child and the child’s mother. We scheduled assessments around each child’s eating and sleeping patterns to prevent hunger or tiredness from affecting results. If the child appeared tired, restless or upset, we rescheduled the testing within the same week to avoid any confounding factors. Before commencing the assessment, we allowed time for the child to acclimate to the testing situation and venue.

### Assessment of gross motor neurodevelopment: Bayley Scales of Infant and Toddler Development, Third edition

The gross motor subscale of the BSID-III (Bayley 2006) was used to assess the gross motor development of children. The raw gross motor developmental scores were converted into age-standardised scaled scores using conversion tables from the BSID-III manual. The scaled scores ranged from 1 to 19. The average score was 10, and the standard deviation was 3. The scaled scores were then classified into three performance categories, namely, above average (≥ 13), average (7–12) and below average (< 7).

### Assessment of gross motor neurodevelopment: Alberta Infant Motor Scale

The AIMS evaluates the development of gross motor skills through 58 items divided into four groups, namely prone (21 items), supine (9 items), sitting (12 items) and standing (16 items). The raw score of each of the four groups is calculated by totalling all observed gross motor items and the preceding gross motor items that are deemed mastered. The AIMS total score is calculated by adding all four group scores together, with possible scores ranging from 0 to 58. This raw score can be adapted to percentile ranks and *z*-scores based on the infant’s age, with the 5th and 10th percentiles serving as cut-off points for delayed gross motor development. The gross motor items are based on three motor control components, such as posture, weight-bearing and antigravity movements. The AIMS is an observational assessment tool with a focus on the spontaneous movement patterns of the infant. Therefore, examiners should avoid handling or actively facilitating movements. However, toys may be used to encourage infants to move between prone, supine and sitting positions.

### Statistical analysis

The Statistical Analysis System (SAS) 9.4 was used for the statistical analysis.

The validity of the AIMS and the gross motor subscale of the BSID-III at 6 months for predicting gross motor developmental delays on the BSID-III at 18 months (scoring < 7) was assessed by using the 10th percentile cut-off score for the AIMS and a scaled score of < 7 for the BSID-III. The Bland-Altman plot was used to determine the agreement between the AIMS and the gross motor subscale of the BSID-III at 6 months. A Bland-Altman plot gives a graphical presentation of the differences between two outcome measures for individual scores and measurement bias (outliers) plotted against the average of the two measures. Furthermore, the receiver operating characteristic (ROC) curves were utilised to determine the predictive ability of the AIMS at 6 months. An area under the curve (AUC) of 1.0 denotes a test with perfect discrimination to detect gross motor outcome, > 0.8 indicates acceptable discrimination, while 0.50 indicates no discriminating ability (Hoo, Candlish & Teare 2017).

### Ethical considerations

The Health Research Ethics Committee of Stellenbosch University’s Faculty of Medicine and Health Sciences granted and approved the current study (reference no: S12/04/111). Permissions were secured from Stikland Psychiatric Hospital and the Western Cape Provincial Health Research Committee in Cape Town, South Africa. Written informed consent was obtained by trained research assistants from the mothers of the participating children in their chosen language, that is, Afrikaans, isiXhosa or English. Mothers were informed that opting out of the study at any point would not negatively affect the quality of care provided to their infants.

## Results

At 6 months, 118 infants were assessed with the gross motor subscale of the BSID-III and the AIMS, while 112 toddlers were evaluated with the BSID-III at 18 months. The six toddlers that were lost to follow-up were excluded from the analysis. At the 6 months assessment, 11 (9.3%) infants scored below average (< 7) on the BSID-III, 7 (6.7%) infants scored below the 10th percentile and 4 infants (3.39%) scored below the 5th percentile on the AIMS. At 18 months of age 11 (9.8%) toddlers scored below average (< 7) on the BSID-III.

**FIGURE 1:**
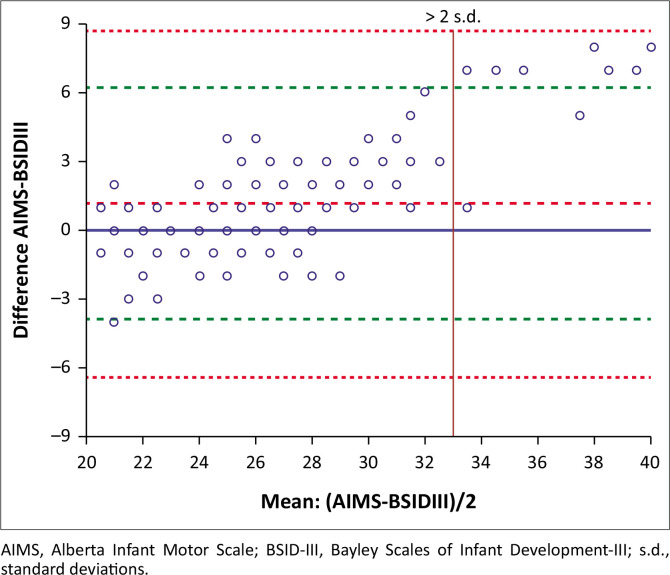
The Bland and Altman scatter plot depicts the agreement between the Alberta Infant Motor Scale and the Bayley Scales of Infant Development-III at 6 months. The solid horizontal blue line indicates the line of no difference between the Alberta Infant Motor Scale and the Bayley Scales of Infant Development-III, the broken red line indicates the mean difference, the green lines indicate 2*x* the standard deviation for the mean difference and the red dotted lines indicate 3*x* the standard deviation of the mean difference (differences that are unacceptable).

Figure 1 indicates the individual differences between the AIMS and BSID-III (*y*-axis) against the mean of the AIMS and BSID-III (*x*-axis). On average, the AIMS values were higher than the BSID-III values. Most of the differences between the two measures are within two standard deviations (s.d.) over the range of values, except for values larger than 33. This indicates a strong agreement between the lower values of the AIMS and the BSID-III scores.

Using the 10th percentile as the AIMS cut-off score at 6 months to identify toddlers scoring below average (< 7) on the BSID-III at 18 months resulted in a sensitivity of 27.3% (95% confidence interval [CI]: 6.0–61.0), a specificity of 98% (95% CI: 92.8–99.8), a positive predictive value (PPV) of 60% (95% CI: 14.7–94.7) and a negative predictive value (NPV) of 92.3% (95% CI: 85.4–96.6).

**FIGURE 2:**
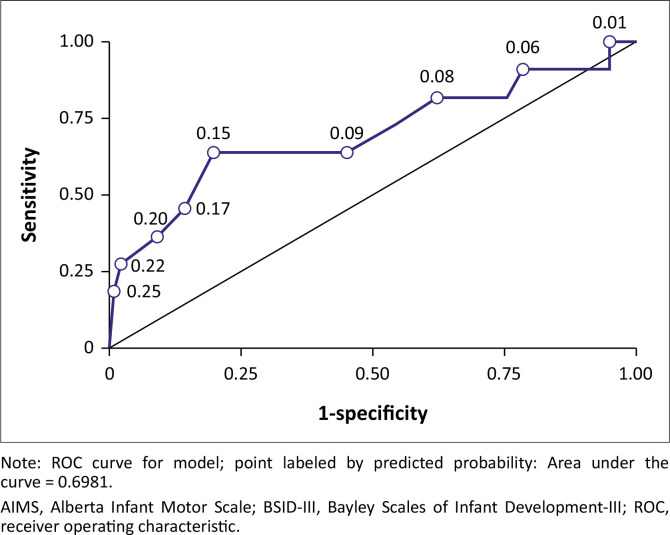
The predictive validity of the Aberta Infant Motor Scale at 6 months to determine gross motor outcome on the Bayley Scales of Infant Development-III at 18 months.

Therefore, the ROC curve analyses were conducted to evaluate if a custom cut-off score tailored to our sample would improve the discriminative ability of the AIMS (Figure 2) at 6 months to identify toddlers scoring below average (< 7) on the BSID-III at 18 months.

Utilising the ROC curve, an optimal AUC of 0.698 (95% CI: 0.494–0.902, *p* = 0.057) for the AIMS (Figure 2) was achieved when the 23rd percentile was used as a cut-off score. The 23rd percentile resulted in a sensitivity of 63.6% (95% CI: 35.2–92.1), a specificity of 81.6% (95% CI: 74.0–89.3), a PPV of 28% (95% CI: 10.4–45.6) and an NPV of 95.2% (95% CI: 90.7–99.8).

## Discussion

Early gross motor developmental delays could be the first or most obvious sign of a broader developmental disorder (Noritz & Murphy 2013). Therefore, the validity of infant neurodevelopmental outcome measures is important for predicting future outcomes and making informed decisions about early intervention aimed at optimising developmental outcomes for at-risk infants, while also providing essential guidance to families during the first year of life. The first aim of the study was to determine the agreement between the AIMS and the BSID-III gross motor domain at 6 months. The Bland-Altman analysis indicated a strong agreement between lower values of the two outcome measures, indicating that the AIMS and the gross motor domain of the BDID-III can be used interchangeably. Bias between the two measures was only detected in the upper-performance range with scores higher than 33. This indicates that AIMS can be used to assess the gross motor behaviour for infants in the lower-performance range but is questionable for larger values above 33. Achieving accuracy in the lower-performance range is crucial when conducting developmental screenings to identify infants who are at risk of delayed gross motor development. This finding is particularly relevant for clinical practice, as early identification of developmental delays typically focuses on identifying infants performing below age-expected norms rather than those showing advanced motor development. Furthermore, the strong agreement between AIMS and BSID-III in the lower-performance range supports the use of the AIMS as a more time-efficient and cost-effective screening tool for identifying infants who may require further comprehensive assessment.

The second aim of our study was to assess the validity of the AIMS at 6 months in predicting severe developmental delay in the BSID-III gross motor domain at 18 months. We found a very low sensitivity of 27.3% (the ability to accurately determine the percentage of infants with gross motor delays), a high specificity of 98% (the ability to accurately determine the percentage of infants without gross motor delays), a PPV of 60% and an NPV of 92.3% when using the 10th percentile as the AIMS cut-off score. Sensitivity and specificity share an inverse relationship, meaning that as one measure increases, the other will decrease (Monaghan et al. 2021). Previous studies also reported a low sensitivity of 42.9% and 50%, respectively, when using a cut-off score of the 10th percentile on the AIMS at 3–5 months to predict gross development on the BSID-III at 9–12 months (Legros et al. 2020) and at 22–24 months (Legros, Zaczek & Mostaert 2023) corrected age in a group of preterm born (< 32 weeks of gestation and/or with a birthweight < 1500 g) infants. These authors suggested using wider cut-off scores namely the 25th percentile when using the AIMS as a screening test to decrease the risk of failing to identify infants at risk of minor gross motor neurodevelopmental delays. We proposed a cut-off score of the 23rd percentile for the AIMS after ROC analysis to predict neurodevelopmental delay (sensitivity of 63.6%, a specificity of 81.6%, a PPV of 28% and an NPV of 95.2%) in a group of full-term infants with a lower risk of neurodevelopmental delays.

Lefebvre et al. (2016) reported that the AIMS 5th centile performed at 4 months corrected age had a sensitivity of 78% and a specificity of 48% for predicting motor delay on the BSID-III assessment at 18 months of age in a group of high-risk extremely preterm infants. The higher sensitivity at this lower cut-off is likely because of the study’s focus on a homogenous group of extremely preterm infants (born at < 28 weeks’ gestation), allowing for a more precise identification of the at-risk population. A recent large meta-epidemiological study analysing 92 meta-analyses published by the Cochrane Collaboration found that as the prevalence of a condition increases, the sensitivity of a diagnostic test also increases. Conversely, as prevalence increases, the specificity of the diagnostic test tends to decrease (Murad et al. 2023).

The accuracy of a screening test can be effectively evaluated using two key statistical measures namely sensitivity and specificity (Monaghan et al. 2021). The main purpose of sensitivity and specificity is to determine if the results of the screening test correspond to the results of the reference gold standard. In the current study, we have therefore assessed if the results of the AIMS at 6 months correspond to the results of the BSID-III at 18 months. Hence, the predictive ability of the AIMS is being assessed. However, for clinicians, it is of more value to predict if an infant truly has a gross motor impairment based on a positive or negative screening test result. Since PPV (the probability that an infant has a gross motor impairment) and NPV (the probability that an infant does not have a gross motor impairment) provide information about the study cohort and not the screening test, they are more relevant than sensitivity and specificity when young infants are being screened (Trevethan 2017). While high sensitivity and specificity are valuable attributes for outcome measures, these metrics should not be the primary basis for decision-making about infants in screening scenarios. Instead, when evaluating screening results for specific patients, clinicians should prioritise the use of PPVs and NPVs, as these provide more relevant information for individual cases (Monaghan et al. 2021; Trevethan 2017).

The findings from our analysis of the predictive validity of the AIMS at 6 months highlight important considerations for its clinical application in gross motor developmental screening. While the relatively low sensitivity at the traditional 10th percentile cut-off point presents a limitation, the high specificity and notably high NPV suggest that the AIMS can effectively identify infants who are unlikely to develop severe motor delays by 18 months. Our proposed adjusted cut-off score at the 23rd percentile offers a more balanced approach, improving sensitivity while maintaining acceptable specificity. This balanced threshold, combined with its high NPV, has positive implications for resource allocation and early intervention planning, as clinicians can be reasonably confident that infants scoring above the 23rd percentile cut-off are likely not to present with gross motor developmental delays. This modification may be particularly valuable in general population screening programmes where the goal is to minimise false negatives while maintaining reasonable efficiency in referral rates. However, the relatively low PPV indicates that positive screening results should be interpreted with caution and infants should be followed up at regular intervals. These results underscore the importance of viewing the AIMS as part of a broader developmental screening strategy rather than as a definitive predictive tool, particularly in populations with varying risk levels for developmental delays.

While this study provides valuable insights into the agreement and predictive validity of the AIMS and the BSID-III for assessing early gross motor development, several limitations should be acknowledged. Firstly, the study sample consisted of full-term infants with a low risk for gross motor delays. Although this ensures a well-defined cohort, it may limit the generalisability of the findings to preterm-born infants or those with high-risk conditions. Future research should explore the applicability of these findings to broader and more diverse populations. Secondly, differences in infant care practices, such as the duration of back carrying or tummy time, were not accounted for, and these factors may have influenced gross motor development. Thirdly, six toddlers were lost to follow-up at 18 months, which may have introduced attrition bias. Although the number is small, missing data could influence the study’s sensitivity and specificity estimates. Despite these limitations, the study contributes valuable evidence supporting the AIMS as a cost-effective screening tool for gross motor development in resource-limited settings. When used in this context, the AIMS can serve as a valuable initial screening instrument that helps guide clinical decision-making while acknowledging the need for ongoing monitoring and potential follow-up assessments.

## Conclusion

The AIMS demonstrates a strong agreement in the lower-performance range with the BSID-III in assessing gross motor development at 6 months, making it a viable screening tool for identifying potential gross motor developmental delays. While the traditional 10th percentile cut-off shows limited sensitivity for predicting motor delays at 18 months, our proposed 23rd percentile threshold offers an improved balance between sensitivity and specificity, with a notably high NPV. These findings underscore the importance of considering both statistical performance metrics and practical clinical utility when implementing developmental screening protocols. Our findings support the use of AIMS as an initial screening instrument, particularly in general population settings. However, clinicians should remain mindful that these predictive properties may vary across different populations and risk groups, and that the AIMS should be used as part of a comprehensive clinical assessment rather than as a standalone diagnostic tool. The AIMS could be integrated into clinical practice, considering its strengths in ruling out motor delays while acknowledging its limitations in definitively predicting them.
